# Impact of semirefined glycerin as an alternative energy source on intake, digestibility and milk production of lactating cows

**DOI:** 10.5713/ab.25.0319

**Published:** 2026-08-25

**Authors:** Murilo de Almeida Meneses, Robério Rodrigues Silva, Dicastro Dias de Souza, Eli Santana de Oliveira Rodrigues, Wêndel Bispo Santos e Santos, Lara Maria Santos Brant, José Esler de Freitas, Fabiano Ferreira da Silva

**Affiliations:** 1 Department of Ruminant Production, State University of Southwestern Bahia, Itapetinga, Brazil; 2 Department of Animal Science, Federal University of Bahia, Salvador, Brazil

**Keywords:** Alternative Feeds, Animal Nutrition, Biodiesel, Glycerol, Lactating Cow

## Abstract

**Objective:**

This study aimed to evaluate the effect of dietary glycerin levels on feed intake, nutrient digestibility, milk yield and composition, nitrogen balance, microbial production, and eating behavior in confined lactating cows.

**Methods:**

Eight crossbred cows (Holstein×Zebu) with an average of 90±10 days in milk, milk production of 21.3±0.47 kg/day, and body weight of 570±51 kg were used. The cows were assigned to two 4×4 Latin squares with the following treatments: Control (G0), no glycerin inclusion; G6, 6% glycerin inclusion in the total dietary dry matter (DM); G12, 12% glycerin inclusion; and G18, 18% glycerin inclusion.

**Results:**

Glycerin inclusion linearly decreased DM intake (p<0.001), crude protein (CP) intake (p = 0.004), and neutral detergent fiber intake (p<0.001). On the other hand, the digestibility coefficients of DM (p = 0.001), CP (p = 0.007), non-fiber carbohydrates (p< 0.001), and total digestible nutrients (p = 0.013) increased linearly. Milk yield (p = 0.046) and 3.5% fat-corrected milk (p = 0.013) decreased linearly.

**Conclusion:**

Despite increasing the digestibility of nutrients, the inclusion of glycerin in diets for feedlot dairy cows resulted in a significant reduction in DM intake, which compromised total energy intake and, consequently, milk production. Therefore, while semi-refined glycerin is an attractive byproduct because of its availability and energy content, it should be used with caution as an energy replacement in the diets of lactating cows at the proportions studied so as not to impair their productivity.

## INTRODUCTION

The intensification of dairy farming has increased challenges related to sustainability, production costs, and the availability of conventional inputs, such as corn and soybeans [1]. In this scenario, the search for nutritional alternatives, which associates productive efficiency with the reduction of environmental impacts, is no longer a trend and has become a strategic necessity for maintaining the competitiveness of the sector. The use of agro-industrial co-products in ruminant feed emerges as a promising alternative [2], not only because it adds value to waste that was previously discarded, but also because it contributes to reducing the environmental impact of the activity and because of its ability to partially replace traditional energy ingredients, often of high cost and with high instability in the market [3].

Among the available co-products, crude glycerin, from the process of transesterification of vegetable oils or animal fats in the production of biodiesel, stands out as a promising alternative in animal feed, especially ruminants [4]. It is estimated that for every 100 kg of biodiesel produced, approximately 10 kg of glycerin are generated [5], which ensures its wide availability on the market and a lower acquisition cost for use in animal feeding. Rich in glycerol (60%–80%) [6], glycerin has a high energy value and acts as a precursor of gluconeogenesis in the liver of ruminants, favoring an improvement in the efficiency of energy metabolism [7].

In recent years, studies have advanced in the search for purer and safer forms of this co-product, culminating in the use of semi-refined glycerin, which has an average glycerol content of 86.95%. This form contains significantly lower concentrations of undesirable impurities, such as methanol, soaps, metallic ions, and pigments, commonly found in crude glycerin [8], allowing its inclusion at higher levels in diets without compromising animal health or performance. Moreover, the higher glycerol concentration provides semi-refined glycerin with superior energy value, promoting its more efficient utilization as an energy source in animal production systems.

Although the benefits of glycerin are recognized, there is a paucity of in-depth studies on the use of semirefined glycerin in feedlot dairy cows, particularly with regard to nutrient digestibility, milk yield, protein metabolism, and eating behavior. Thus, we hypothesize that the inclusion of different levels of semi-refined glycerin in the diet of confined dairy cows does not cause significant changes in milk production, indicating that glycerin can be used as a viable and safe alternative feed without compromising the productive performance of the animals. Based on this premise, this study aimed to evaluate the effects of the inclusion of different levels of glycerin in diets for confined dairy cows, analyzing their impact on nutrient intake and digestibility, milk production and composition, nitrogen balance, microbial protein synthesis and eating behavior.

## MATERIAL AND METHODS

### Ethics and Localization Committee

All experimental procedures were carried out with the approval of the Animal Ethics Committee under protocol 13/012 of the State University of Southwest Bahia, by national and international guidelines. During the experiment, cows were housed in a barn containing individual pens (19 m^2^), sand beds, feed bunks, and free access to water. The experiment was carried out on a commercial farm, located in the municipality of Encruzilhada-BA, under the coordinates: latitude 15° 31′ 49″ South, longitude 40° 54′ 37″ West, at an altitude of 915 meters.

### Animals, experimental design and diets

Eight crossbred cows (Holstein×Zebu) (mean days in lactation = 90±10 days; milk production = 21.3±0.47 kg/day; body weight = 570±51 kg) were distributed in two 4×4 Latin squares, with four experimental periods. Each period lasted 21 days, with the first 16 days for adaptation and 5 days for data collection. The animals were weighed every 21 days after milking to assess body weight. The cows were randomly fed four experimental diets:

G0 = control (without the inclusion of glycerin in the diet);
G6 = inclusion of 6% of glycerin in the dry matter (DM) of the diet;
G12 = inclusion of 12% of glycerin in the DM of the diet; and
G18 = inclusion of 18% of glycerin in the diet’s DM.

The diets were formulated according to the National Research Council (NRC) [9] to meet the nutritional needs of lactating cows with an average milk production of 25 kg/day.

The glycerin was acquired from Aboissa Commodity Brokers. It has 703.4 g/kg of DM, 920.6 g/kg of DM of organic matter (OM), 904.6 g/kg of DM of glycerol, 28.8 g/kg of DM of lipids, 0.2 g/kg of DM of methanol, density of 1.26 g/cm^3^ and viscosity of 336.50 cps.

Table 1 presents the ingredient proportions and the chemical-bromatological composition of the formulated experimental diets. The formulation was carefully adjusted to partially replace traditional energy ingredients, such as ground corn, with glycerin, taking advantage of its high glycerol content and energy value. To maintain an adequate nutritional balance, it was necessary to adjust the proportions of corn silage and soybean meal, ensuring a balanced supply of protein. Table 2 presents the chemical composition of the main ingredients used in the experimental diets.

### Nutrient intake and apparent digestibility of the total tract

The animals were fed with equal amounts twice a day (07:00 and 15:00), aiming for leftovers between 50 and 100 g/kg. Between the 17th and 21st day of each experimental period, samples of corn silage, concentrated ingredients and leftovers were collected and stored at −20°C for further analysis. The samples of leftovers were collected daily and, composite animal/period sampling was performed after the collection period.

On days 18, 19, and 20 of each experimental period, fecal samples were collected at 9-hour intervals, either directly from the rectum or immediately after defecation. After collection, the samples were preserved and, a composite sample was made for each animal at the end of each period. The total fecal excretion for each animal was determined based on the concentration of indigestible neutral detergent fiber (_i_NDF) used as an internal marker, following the recommendations of Casali et al [10].

### Sample collection and laboratory analysis

At the beginning of the experiment, samples of crude glycerin were analyzed to evaluate DM (ASTM [11], method 6304.07), ash (AOAC International [12], method 920.39), and crude protein (CP, AOAC International [12]; method 984.13). The glycerol, methanol and lipid contents were determined using an Agilent Technologies gas chromatograph (GC 7890A) equipped with an electron impact ionization detector (MS 5975C) and DB-5MS capillary column (Agilent Technologies, 30 m long×0.25 mm internal diameter×0.25 μm film thickness).

Samples of corn silage, concentrated ingredients, feed refusals and feces were dried in a forced air circulation oven (55°C for 72 h) and subsequently ground to 1 mm and 2 mm for further analysis.

Samples at 1 mm were analyzed for DM (method 930.15; AOAC International [12]), ash (method 942.05; AOAC International [12]), CP (N×6.25; Kjeldahl Method 984.13; AOAC International [12]), ether extract (EE, method 920.39; AOAC International [12]). Neutral detergent fiber (NDF) was analyzed according to Mertens [13], using thermosetting alpha-amylase without the addition of sodium sulfite to the detergent in a unit ANKOM200 Fiber Analyser (Ankom Technology) and the starch content was analyzed according to Hall [14]. Metabolizable energy (ME) was estimated according to NRC [9]. The _i_NDF was determined for the 2 mm samples according to Casali et al [10].

### Nitrogen balance and excretion of purine derivatives

The urine was collected between the 18th and 20th day of each experimental period, every 9 hours, through spot collection (50 mL). A subsampling was performed for storage, with a portion stored at −20°C, and 10 mL of urine was stored diluted in 40 mL of 0.036 N sulfuric acid.

Creatinine (ID: K067), uric acid (ID: K139), and urea concentrations in urine and deproteinized milk were determined using Bioclin commercial kits (Bioclin). The concentration of allantoin in the urine was analyzed according to Fujihara et al [15]. The total excretion of purine derivatives (SD; mmol/day), absorbed purine derivatives (absP, mmol/day) were determined according to Chen and Gomes [16].

Each animal’s daily urine volume was quantified by multiplying individual body weight by the daily creatinine excretion rate and dividing the result by the creatinine concentration (mg/L) in the urine sample. A daily creatinine excretion rate of 24.05 mg/kg of body weight was assumed, as proposed by Chizzotti et al [17]. Nitrogen balance (BN) was calculated using nitrogen intake and the amounts of nitrogen excreted in animal feces, urine, milk, and feed (ID: 984.13; AOAC [12]).

### Blood metabolites

Blood samples were collected on the 21st day of each period, obtaining 10 mL through puncture of the coccygeal artery or vein, only once, always at 08:00 h. Immediately, the tubes were centrifuged (1,800×g for 15 min) to separate the plasma. After centrifugation, the plasma was kept in Eppendorf tubes under refrigeration (thermal box with ice), and transported to the laboratory, where it was stored at −20°C for further analysis.

The samples were analyzed for urea nitrogen concentrations using a commercial enzymatic colorimetric kit (Urea – ID: K047, Bioclin) on a semiautomatic biochemical analyzer (AJX-1900; Micronal). The intra-assay coefficient of variation for this method was 2.28%.

### Milk production and composition

The cows were milked twice daily and milk production was monitored using an automatic milking (MMI6). Milk production was corrected to 3.5% fat-corrected milk (FCM), according to Sklan et al [18]. Milk samples were collected for three consecutive days (18 to 20 of each period) to evaluate protein, fat, and lactose concentrations using the mid-infrared method (LACTOSCAN, Entelbras). Feed efficiency (FE) was calculated as the ratio between milk production (kg/day) and dry matter intake (DMI) (kg/day).

### Eating behavior

Eating behavior was evaluated on the 17th day of each experimental period. Individual observers conducted 24-hour observations at 5-minute intervals to determine the time spent in rumination, feeding, and idleness (where the latter denotes the time spent without performing the other two activities). Two trained observers were positioned to minimize the disturbance in the cows’ behavior. The environment was artificially lit before data collection to help the animals adapt.

In three 2-hour periods throughout the day (morning, afternoon, and night), assessments were made to determine the number of chews per ruminated bolus and the time taken to ruminate this bolus, using stopwatches.

FE (EAL), rumination efficiency (ERU), number of rumen boluses per day (NBR), total chewing time per day (TMR) and number of meric chews per day (NMMnd) were calculated, as proposed by Polli et al [19] and Bürger et al [20].

The voluntary intake of DM and NDF was considered to evaluate the feeding and rumination efficiencies in relation to the amount in grams of DM and NDF per unit of time and feeding period. The number of cakes ruminated daily was obtained by dividing the total rumination time (minutes) by the average time spent ruminating on a cake.

### Statistical analysis

A replicated 4×4 Latin square design balanced for residual effect was used [21]. Intake, gastrointestinal tract digestibility, production parameters, milk composition, nitrogen compound balance, urea nitrogen concentration, microbial protein production and microbial efficiency and eating behavior data were analyzed using the PROC MIXED procedure of Statistical Analysis System software [22] (Statistical Analysis System – ver. 9.4; SAS Institute). The model equation is as follows:


(1) $${\text{Y}}_{ijkl}=\mu +{\text{S}}_{i}+{\text{A}}_{i}{\left(\text{S}\right)}_{j}+{\text{P}}_{k}+{\text{T}}_{l}+{\text{e}}_{ijkl}$$

Where Y*_ijkl_* is the dependent variable, *μ* is the overall mean, S*_i_* is the fixed effect of Latin square *i* (cow group) (*_i_* = 1–2), A*_j_* (S*_i_*) is the random effect of animal within square *_j_* (*_j_* = 1–8), P*_k_* is the fixed effect of experimental period *_k_* (*_k_* = 1–4), T*_l_* is the fixed effect of treatment *_l_* (*_l_* = 1–4) and e*_ijkl_* is the residual error.

Orthogonal contrasts were used to evaluate semirefined glycerin, linear (−3 −1 1 3), quadratic (1 −1 −1 1) and cubic (−1 3 −3 1) effects of the inclusion of semirefined glycerin. Statistical differences were considered significant at p≤0.05 and trends were considered when 0.05<p≤0.10.

## RESULTS

The inclusion of different levels of glycerin as a feed additive promoted a linear decrease in DMI (%BW) (p<0.001), NDF (%BW) (p<0.001), DM (kg/day) (p<0.001), CP (kg/day) (p=0.004), and NDF (kg/day) (p<0.001) (Table 3). There was a tendency for a decreasing linear effect on total digestible nutrients (TDN) intake (p = 0.085). In contrast, the intake of EE and NFC remained unchanged by glycerin inclusion (p>0.05).

Regarding digestibility, glycerin caused a linear increase in total digestibility of DM (p = 0.001), CP (p = 0.007), NFC (p<0.001), and TDN (p = 0.013). There was also a tendency for a linear increase in EE digestibility with glycerin inclusion (p = 0.056).

A linear decrease was observed in milk yield (p = 0.046), FCM (3.5%) (p = 0.013), fat yield (kg/day) (p = 0.014), and total solids yield (kg/day) (p = 0.025) with increasing inclusion of glycerin in the diet (Table 4). There was also a trend toward reduced lactose yield (kg/day) (p = 0.062), solid non-fat yield (kg/day) (p = 0.074), and FE (p = 0.097). However, the percentage composition of milk components was not affected by glycerin inclusion in the experimental diets (p>0.05).

Nitrogen-related parameters indicated that glycerin inclusion resulted in a linear reduction in N intake (p = 0.004) and fecal N excretion (p<0.001) (Table 5), without affecting milk, urine, retained, or digested nitrogen (p>0.05). Moreover, plasma and milk urea nitrogen concentrations remained stable (p>0.05). A tendency for a linear decreasing effect was observed for total allantoin excretion (p = 0.066) and total purine derivatives (p = 0.064). No significant changes were observed in microbial efficiency expressed as grams of CP per kilogram of ingested TDN (p>0.05).

Glycerin inclusion did not affect the time spent on feeding, rumination, and idling activities (hours/day) (p>0.05). However, it caused a linear reduction in NDF feeding efficiency (kg NDF/h) (p<0.001), DM rumination efficiency (kg DM/h) (p = 0.001), and NDF rumination efficiency (kg NDF/h) (p<0.001) (Table 6). DM feeding efficiency (kg DM/h) was not affected (p>0.05).

Glycerin had no influence on TCT (h/day), NBR (nº/day), NCd (nº/day), NCb (nº/day), and TSB (sec/bolus) (p>0.05). Likewise, the number and duration of daily feeding, rumination, and idling periods were not affected by the treatment (p>0.05).

## DISCUSSION

The animals fed glycerin showed a reduction in DMI. The literature is inconsistent regarding the relationship between dietary intake and glycerol supplementation [23]. As in the present study, some researchers have observed that feed intake decreases as the dose of glycerol increases in ruminants [24]. This effect may be related to the purity of the glycerol and the proportion absorbed directly compared to that fermented in the rumen. Glycerol is a glycogenic precursor that can be absorbed directly from the rumen by passive diffusion and used in gluconeogenesis, or rapidly fermented by rumen microorganisms to produce volatile fatty acids (VFAs), such as propionate and butyrate [25].

Thus, the lower DMI observed in glycerin-fed animals can be interpreted in the light of the hepatic oxidation theory [26], which proposes the sending of inhibitory signals from the liver to the hypothalamus, promoting the feeling of satiety. This effect is related to increased oxidation of gluconeogenic precursors, and increased ATP synthesis in the liver, especially in diets with high glycerol levels.

In addition, the change in the ratio of forage: concentrate, associated with the inclusion of glycerin, as it is a liquid food, modified the physical form of the diet, making it more pasty and viscous [26]. This change may have impaired the mixing of ingredients and compromised palatability. Consequently, there may have been selective rejection of less attractive components, reducing total intake. As a result of the reduction in DMI, CP and TDN intake were also reduced in the glycerin diets. This is because diets have been formulated to be isonitrogenous. In addition, NDF intake (in kg/day and % of body weight) was also reduced due to the lower DMI observed in glycerin-fed animals. In addition, the lower consumption of NDF reflects the lower concentration of this nutrient in diets with glycerin, since the by-product is free of fiber.

However, an important contribution of this study was the increase in the digestibility of DM, CP, NFC and the tendency to increase the digestibility of EE with the inclusion of glycerin. By replacing corn starch with glycerol present in glycerin, there is an increase in the digestion of NFC and, consequently, in the digestibility of DM. This is because the digestion of glycerol is faster than that of corn starch, due to its structural characteristics and its mechanism of metabolization in the rumen. Glycerol is a simple compound, with low molecular weight and highly soluble in water, which facilitates its rapid passive diffusion through the rumen wall and its direct use in gluconeogenesis [27]. In addition, when it is not absorbed directly, glycerol is rapidly fermented by rumen microorganisms, generating VFA, mainly propionate, more efficiently. On the other hand, corn starch, a complex polysaccharide, requires enzymatic hydrolysis prior to microbial fermentation. This process depends on the action of amylases and is relatively slower compared to glycerol.

Another relevant factor is that the lower DM intake observed in animals fed with glycerin implies a reduction in the rate of passage of the diet, so that the food particles remain longer in the rumen, allowing a greater extension of fermentation and, consequently, increasing the digestibility of DM, as well as non-fiber carbohydrates and CP. It is worth noting that, in addition to the longer permanence time of the diet in the digestive tract, the diets with glycerin had a higher content of soybean meal, an ingredient with high digestibility of the protein fraction.

The increased extract digestibility may be related to the greater supply of fatty acids from glycerin in the small intestine, which favors micelle formation and lipid absorption [28]. Additionally, this improvement may result from the dilution effect of endogenous lipid losses in the feces, leading to a higher digestibility coefficient [29]. This digestive effect was accompanied by an increase in the digestibility of TDN, associated with an increase in the digestibility coefficients of DM, CP, NFC and EE trend, which resulted in greater utilization of nutrients from the diet.

Despite the increase in nutrient digestibility, the increasing inclusion of glycerin resulted in a linear reduction in milk production, FCM (3.5%), and in fat and total DM production. This effect is directly related to lower IMD, the main determinant of energy and nutritional intake in high-producing cows [26]. Thus, the reduction in consumption compromised the energy supply necessary for the synthesis of milk components.

In addition to the lower DMI, the partial replacement of ground corn by glycerin reduced the concentration of starch in the diet. Starch is the main fermentable carbohydrate in the feed, being rapidly degraded in the rumen, with predominant production of propionate, a volatile fatty acid that acts as the main precursor of hepatic glucose and, consequently, lactose, responsible for the osmotic regulation of milk volume [9]. Although the glycerol present in glycerin can be converted to glucose, its direct absorption by the rumen wall limits its fermentation and thus reduces conversion to propionate [30].

Propionate is responsible for approximately 60%–74% of hepatic gluconeogenesis in lactating cows [31], and is recognized as the most efficient and physiologically dominant substrate in this process. Recent studies demonstrate that propionate stimulates the expression of key gluconeogenic genes, contributing significantly to glucose production [32].

Despite the fact that glycerol is rapidly absorbed and converted to glycerol-3-phosphate, entering directly into the gluconeogenic pathway [33], propionate remains the main gluconeogenic substrate in lactating cows. Thus, the alteration in the fermentation profile, associated with the lower availability of gluconeogenic substrates, may have compromised lactose synthesis and, consequently, milk production.

The reduction of milk fat (kg/day) in diets with glycerin is associated with lower intake of DM and DNF, which compromise rumen fermentation and decrease the production of acetate and butyrate, essential precursors for lipid synthesis in the mammary gland [34], resulting in reduced fat synthesis.

In addition, although the percentage composition of milk remained stable, the absolute production of solids was compromised by the lower energy intake and the lower supply of specific precursors, reinforcing the impact of feed intake on total productivity.

The inclusion of glycerin in the diets also reduced nitrogen intake, accompanied by lower fecal excretion of the nutrient. Because the diets were formulated to be isonitrogenous, this reduction is mainly associated with the drop in DMI in glycerin treatments. The lower fecal excretion may reflect not only the reduced intake, but also a possible improvement in protein digestibility, potentially due to the longer rumen retention time, which favors the action of digestive enzymes on dietary nitrogen.

Despite the lower nitrogen intake, the amount absorbed was sufficient to maintain a positive nitrogen balance in all treatments, indicating that the available nitrogen met the metabolic demands of the animals, being used in body protein synthesis, tissue renewal and maintenance of essential physiological functions [35]. As a result, there was no weight loss or reduction in productivity associated with protein deficiency, showing that protein requirements were fully met.

Purine derivatives, especially allantoin, are widely used as indirect indicators of microbial protein synthesis in the rumen [16]. The reduction in excretion of these compounds suggests a possible decrease in the absolute production of microbial protein with the inclusion of glycerin. However, microbial efficiency, expressed in grams of microbial protein per kilogram of TDN ingested, was not affected. This indicates that, despite a possible slight reduction in total production, the efficiency in the use of the energy substrate for microbial synthesis was maintained. Thus, even with a quantitative reduction in microbial synthesis markers, the maintenance of efficiency suggests that ruminal microorganisms continued to use nitrogen effectively, contributing to the proper use of the nutrient.

Therefore, the negative effects observed on the productive performance of cows fed glycerin are not due to protein limitation, but rather to lower energy intake, reduction of rapidly fermentable carbohydrates, especially starch, changes in the ruminal fermentation profile and metabolic particularities of glycerol. These factors, combined with the lower DMI, limited the supply of energy and metabolic precursors essential to milk synthesis, even in the face of increased nutrient digestibility.

Despite the reduction in milk production with the inclusion of glycerin, there was an upward trend in EF. This result indicates that the animals converted the ingested food more efficiently, even with lower total intake. Such behavior may be associated with the high digestibility of glycerin, which provides energy quickly and efficiently [30]. In addition, the reduction in intake may have increased the rumen retention time, as already mentioned, optimizing the use of nutrients. It is also noteworthy that the use of glycerin can reduce methane production during rumen fermentation, which contributes to greater efficiency in the use of ME [36].

A significant advantage of this study is that, despite the reduction in DM intake in glycerin diets, the time dedicated to feeding was not altered, which reinforces the hypothesis of lower acceptability of glycerin diets due to the change in the physical shape of the diet, making it more viscous and less palatable. This implies a selective activity of the animals, suggesting that the physical form of the diet is a determining factor for its acceptability.

On the other hand, the feed and rumination efficiencies of NDF (EALFDN and ERUFDN) were reduced with the inclusion of increasing glycerin levels in the diets. FE represents the speed of nutrient intake as a function of time, while rumination efficiency is associated with the quantity and quality of food ingested [37]. Thus, the reduction observed in this study reflects the lower amount of NDF to be consumed, due to its lower concentration in diets with glycerin, as well as the lower amount of NDF to be ruminated, precisely because of its lower consumption.

## CONCLUSION

Despite increasing the digestibility of nutrients, the inclusion of glycerin in diets for feedlot dairy cows resulted in a significant reduction in DMI, which compromised total energy intake and, consequently, milk production. Thus, although it is a promising co-product due to its availability and energy value, semi-refined glycerin, in the proportions tested, was not viable as an energy substitute in diets of lactating cows, requiring caution not to compromise productive performance. Therefore, future studies should explore different inclusion levels, degrees of purity, and strategies for combining glycerin with other ingredients, as well as evaluate its effects at different stages of lactation, in order to enable the safe and efficient use of semi-refined glycerin in dairy cow nutrition.

## Figures and Tables

**Table 1:** Ingredient proportions and chemical-bromatological composition of the experimental diets

Ingredients (g/kg of dry matter [DM])	Glycerin levels (% DM)			

	G0	G6	G12	G18
Corn silage	520.0	529.8	534.6	540.0
Ground corn	322.0	235.4	155.8	72.8
Soybean meal	129.8	143.6	156.9	171.8
Glycerin1)	0.00	63.0	124.5	187.2
Mineral2)	11.8	11.8	11.8	11.8
Sodium bicarbonate	5.40	5.40	5.40	5.40
Urea	6.00	6.00	6.00	6.00
Limestone	5.00	5.00	5.00	5.00
Chemical composition (g/kg of DM)				
DM	596.4	580.0	566.7	552.8
Crude protein	154.8	154.8	154.7	155.2
Ether extract	31.9	30.8	29.8	28.8
Neutral detergent fiber	288.1	283.5	277.5	271.6
Starch	37.6	31.6	25.9	20.0
Ash	68.9	73.9	78.6	83.5
Metabolizable energy (ME) (Mcal/kg of DM)3)	2.66	2.86	3.06	3.26

1) Composition: DM 703.4 g/kg of DM; organic matter, 920.6 g/kg of DM; glycerol, 904.6 g/kg of DM; lipids, 28.8 g/kg of DM; methanol, 0.2 g/kg of DM; crude protein, 0.001 g/kg of DM; density, 1.26 g/cm^3^; viscosity, 336.50 cps.

2) Composition: Calcium 200 g; Cobalt 200 mg; Copper 1.650 mg; Sulfur 12 g; Iron 560 mg; Fluorine (max) 1.000 g; Phosphorus 100 g; Iodine 195 mg; Magnesium 15 g; Manganese 1.960 mg; Nickel 40 mg; Selenium 32 mg; Sodium 68 g; Zinc 6.285 mg.

3) ME estimated by the NRC equation [9].

**Table 2:** Chemical composition of the main ingredients used in the experimental diets

Chemical composition (g/kg of dry matter [DM])	Corn silage	Ground corn	Soybean meal
DM	327.2	878.8	886.5
Crude protein	85.0	89.6	500.0
Ether extract	31.8	40.0	19.3
Neutral detergent fiber	444.6	123.9	131.0
Starch	251.5	736.6	61.8
Ash	52.1	16.2	64.7
Metabolizable energy (ME) (Mcal/kg of DM)1)	2.43	2.98	3.37

1) ME estimated by the NRC equation [9].

**Table 3:** Intake and total-tract digestibility of dry matter (DM) and nutrients of lactating cows fed different levels of blonde glycerin in the diet

Items	Glycerin levels (% DM)				SEM	p-value1)	
	
	G0	G6	G12	G18		L	Q
Intake (%BW)							
Dry matter	4.11	3.96	3.84	3.63	0.07	<0.001	0.763
Neutral detergent fiber	1.48	1.32	1.23	1.10	0.03	<0.001	0.582
Intake (kg/day)							
Dry matter	23.38	22.55	21.78	20.54	0.53	<0.001	0.687
Crude protein	3.05	3.02	2.75	2.74	0.07	0.004	0.911
Neutral detergent fiber	8.42	7.52	6.99	6.22	0.23	<0.001	0.680
Ether extract	0.62	0.70	0.62	0.67	0.02	0.734	0.678
Non-fibrous carbohydrates	9.67	9.60	9.72	9.23	0.24	0.359	0.472
TDN	17.22	17.09	16.92	16.10	0.38	0.085	0.439
Total-tract digestibility							
Dry matter	65.35	67.59	69.42	70.93	0.68	0.001	0.742
Crude protein	70.99	71.34	71.84	76.53	0.86	0.007	0.104
Neutral detergent fiber	53.89	51.06	52.07	50.51	1.33	0.301	0.743
Ether extract	76.94	82.63	80.85	85.01	1.44	0.056	0.760
Non-fibrous carbohydrates	81.25	84.92	88.15	89.49	0.80	<0.001	0.294
TDN	65.85	67.53	69.30	70.88	0.68	0.013	0.960

1) L = linear effect of GLY; Q = quadratic effect of GLY (statistical differences were considered significant at p≤0.05 and trends were considered when 0.05<p≤ 0.10).

SEM, standard error of the means; TDN, total digestible nutrients.

**Table 4:** Productive parameters and milk composition of lactating cows fed different levels of blonde glycerin in the diet

Items	Glycerin levels (% DM)				SEM	p-value1)	
	
	G0	G6	G12	G18		L	Q
Milk yield (kg/d)	21.29	20.76	20.92	20.11	0.67	0.046	0.711
FCM (3.5%) (kg/d)	27.47	27.17	26.97	25.11	0.79	0.013	0.202
Protein (kg/d)	0.71	0.69	0.69	0.67	0.02	0.119	0.989
Fat (kg/d)	1.12	1.12	1.10	1.01	0.03	0.014	0.150
Lactose (kg/d)	1.06	1.03	1.04	0.99	0.03	0.062	0.659
Minerals (kg/d)	0.16	0.16	0.16	0.15	0.00	0.100	0.862
Defatted dry extract (kg/d)	1.93	1.88	1.88	1.82	0.05	0.074	0.779
Total dry extract (kg/d)	3.05	3.00	2.99	2.83	0.09	0.025	0.387
Composition (%)							
Protein	3.31	3.32	3.31	3.35	0.02	0.462	0.605
Fat	5.34	5.42	5.35	5.08	0.12	0.163	0.181
Lactose	4.98	4.99	4.97	4.94	0.03	0.399	0.741
Minerals	0.77	0.77	0.76	0.77	0.00	0.862	0.897
Defatted dry extract	9.06	9.09	9.05	9.05	0.06	0.805	0.939
Total dry extract	14.40	14.52	14.40	14.14	0.17	0.242	0.261
FE (kg milk/DMI)	1.17	1.20	1.24	1.24	0.02	0.097	0.693

1) L = linear effect of GLY; Q = quadratic effect of GLY (statistical differences were considered significant at p≤0.05 and trends were considered when 0.05<p≤0.10).

DM, dry matter; SEM, standard error of the means; FCM (3.5%), milk yield was corrected to 3.5% fat; FE, feed efficiency; DMI, dry matter intake.

**Table 5:** Balance of nitrogen compounds, urea nitrogen concentration, purine derivatives and microbial efficiency of lactating cows receiving different levels of blonde glycerin in the diet

Items	Glycerin levels (% DM)				SEM	p-value1)	
	
	G0	G6	G12	G18		L	Q
Nitrogen compound balance (g/d)							
Ingested-N	487.92	483.19	440.32	438.52	11.93	0.004	0.911
Fecal-N	184.11	180.29	161.01	131.57	6.63	<0.001	0.126
Milk-N	112.89	107.81	110.43	106.79	3.49	0.119	0.989
Urinary-N	163.86	158.58	145.78	159.38	5.53	0.130	0.583
Retained-N	27.05	36.52	23.09	40.78	10.06	0.373	0.527
Digested-N	303.80	302.90	279.31	306.96	9.65	0.821	0.313
Urea nitrogen concentrations (mg/dL)							
Plasma	13.06	12.21	13.22	12.00	0.35	0.385	0.744
Milk	13.16	11.62	12.45	11.14	0.59	0.119	0.877
Purine derivatives (mmol/d)							
Total allantoin	312.17	309.93	295.39	250.92	16.54	0.066	0.364
Uric acid	9.06	9.05	6.95	7.35	0.68	0.243	0.880
Total purine derivatives	320.85	318.95	302.53	258.28	16.83	0.064	0.373
Microbial efficiency							
g CP/kg TDN intake	79.58	72.71	74.55	87.26	4.95	0.354	0.112

1) L = linear effect of GLY; Q = quadratic effect of GLY (statistical differences were considered significant at p≤0.05 and trends were considered when 0.05<p≤0.10).

DM, dry matter; SEM, standard error of the means; CP, crude protein; TDN, total digestible nutrients.

**Table 6:** Eating behavior of lactating cows receiving different levels of blonde glycerin in the diet

Items	Glycerin levels (% DM)				SEM	p-value1)	
	
	G0	G6	G12	G18		L	Q
Time spent (h)							
Feeding	4.37	4.23	4.35	4.44	0.15	0.729	0.571
Rumination	7.47	7.53	7.71	7.86	0.25	0.373	0.890
Idle	12.16	12.24	11.94	11.70	0.26	0.377	0.701
Feed efficiency							
DM (kg DM/h)	5.46	5.44	5.35	4.92	0.24	0.125	0.399
NDF (kg NDF/h)	1.98	1.82	1.72	1.49	0.09	<0.001	0.620
Rumination efficiency							
DM (kg DM/h)	3.31	3.02	2.90	2.63	0.11	0.011	0.958
NDF (kg NDF/h)	1.18	1.01	0.93	0.80	0.04	<0.001	0.750
TCT (h/day)	11.84	11.76	12.06	12.30	0.26	0.377	0.701
NBR (nº/day)	446	447	491	480	17.9	0.256	0.822
NCd (nº/day)	27060	26808	27178	28156	1025	0.533	0.638
NCb (nº/bolus)	61.57	60.56	55.75	59.11	1.47	0.166	0.261
TSB (sec/bolus)	61.98	61.53	56.68	59.29	1.36	0.133	0.416
Number of periods (number/d)							
Feeding	13.6	12.4	14.88	13.75	0.62	0.397	0.934
Rumination	15.3	17.13	15.63	15.38	0.57	0.806	0.307
Idle	22.8	21.75	23.75	23.63	0.62	0.299	0.656
Time per period (h)							
Feeding	0.33	0.35	0.30	0.33	0.00	0.525	0.966
Rumination	0.50	0.45	0.50	0.53	0.02	0.287	0.222
Idle	0.53	0.58	0.52	0.52	0.02	0.471	0.501

1) L = linear effect of GLY; Q = quadratic effect of GLY (statistical differences were considered significant at p≤0.05 and trends were considered when 0.05<p≤0.10).

DM, dry matter; SEM, standard error of the means; NDF, neutral detergent fiber; TCT, total chewing time; NBR, number of boluses ruminated per day; NCd, number of chewsper day; NCb, number of chews per bolus; TSB, time spent per bolus ruminated.

## Data Availability

Upon reasonable request, the datasets of this study can be available from the corresponding author.
